# Distribution and abundance of Peleng Tarsier (*Tarsius pelengensis*) in Banggai Island group, Indonesia

**DOI:** 10.1038/s41598-023-30049-5

**Published:** 2023-07-15

**Authors:** Fakhri Naufal Syahrullah, Un Maddus, Abdul Haris Mustari, Sharon Gursky, Mochamad Indrawan

**Affiliations:** 1 Department of Conservation of Forest Resources and Ecotourism, Faculty of Forestry and Environment, IPB University, Bogor, 16680 Indonesia; 2 Togong Tanga Indigenous People’s Community, Banggai Island District, Central Sulawesi 94881 Indonesia; 3 Department of Anthropology, MS 4352, Texas A&M University, College Station, TX 77845 USA; 4 Center for Biodiversity and Conservation, Universitas Indonesia, Depok, 16424 Indonesia

**Keywords:** Conservation biology, Biodiversity

## Abstract

The Peleng tarsier (*Tarsius pelengensis*) is poorly known primate, with a range limited to Banggai island-group, Central Sulawesi, Indonesia. It was classified as “Endangered” by IUCN in 2017 based on extremely limited demographic and distributional data. The aim of this study was to collect and analyze data on the population and distribution of Peleng tarsiers. Surveys were conducted over approximately 5 months in 2017 and 2018 across Peleng and the neighboring islands of Banggai, Labobo, and Bangkurung. We determined that tarsiers only occur on Peleng and Banggai Island. The average population density in Peleng and Banggai was estimated to be 234 individuals/km^2^. This is comparable to the broad ranges of tarsier densities throughout Sulawesi and offshore islands. Peleng tarsiers were found in all elevations (0–937 m above sea level) and nearly all vegetated habitats in Peleng island. Using the IUCN criteria for determining conservation status, in conjunction with our new data, we believe that the Peleng tarsier population should be classified as “Vulnerable”.

## Introduction

Tarsiers are found on numerous islands throughout the Philippines, Malaysia, and Indonesia. There are currently 14 species of tarsiers in three genera: *Cephalopachus* in Kalimantan/ Borneo and Sumatera, *Tarsius* in Sulawesi and *Carlito* in the Philippines^1^ Of the 14 tarsier species, 13 are found in Indonesia and 12 of the 13 are exclusively found in Sulawesi Indonesia and its outlying islands.

The Peleng tarsier is found only on Peleng Island, off the isthmus of Central Sulawesi^1–7^. Peleng is the largest island of the Banggai Island group, which also includes Banggai, Labobo, and Bangkurung. Prior to this study, it was not known whether Peleng tarsier existed on the surrounding islands.

While preliminary natural history information about the Peleng tarsier has been collected, demographic and distributional data about the Peleng tarsier are lacking. Given the importance of this information for establishing conservation priorities and strategies, the goal of this study is to obtain this information and to collect and analyze the distribution, population, density and habitat use of the Peleng tarsier along with re-evaluating its conservation status.

## Materials and methods

### Study site and subjects

The Banggai archipelago (Peleng I, Banggai I, Labobo I, and Bangkurung I) is vegetated by a mosaic of cultivation and scrubland with remnant natural ecosystems comprised of dryland tropical moist forests, mangroves, beach forests (Table 1).

**Table 1:** Comparison between area and forest cover in study site.

Island	Size (Km^2^)	Forest cover remaining* (Km^2^)	Types of forest
Peleng Island	2340	1172.9	Dryland tropical moist forest, mangroves, and beach forest
Banggai Island	280	165.7	Dryland tropical moist forest, mangroves, and beach forest
Labobo Island	73	55.8	Dryland tropical moist forest, mangroves, and beach forest
Bangkurung Island	118	109.9	Dryland tropical moist forest, mangroves, and beach forest

*Extent estimated by the authors from Republic of Indonesia Geospatial Information Agency 2018 and Global Forest Watch 2018.

A previous study^8^ on Peleng and surrounding islands found multiple endemic animals with threatened or near-threatened conservation status. Local wildlife population size was reduced by habitat degradation, predators, and trapping^8^.

**Figure 1:**
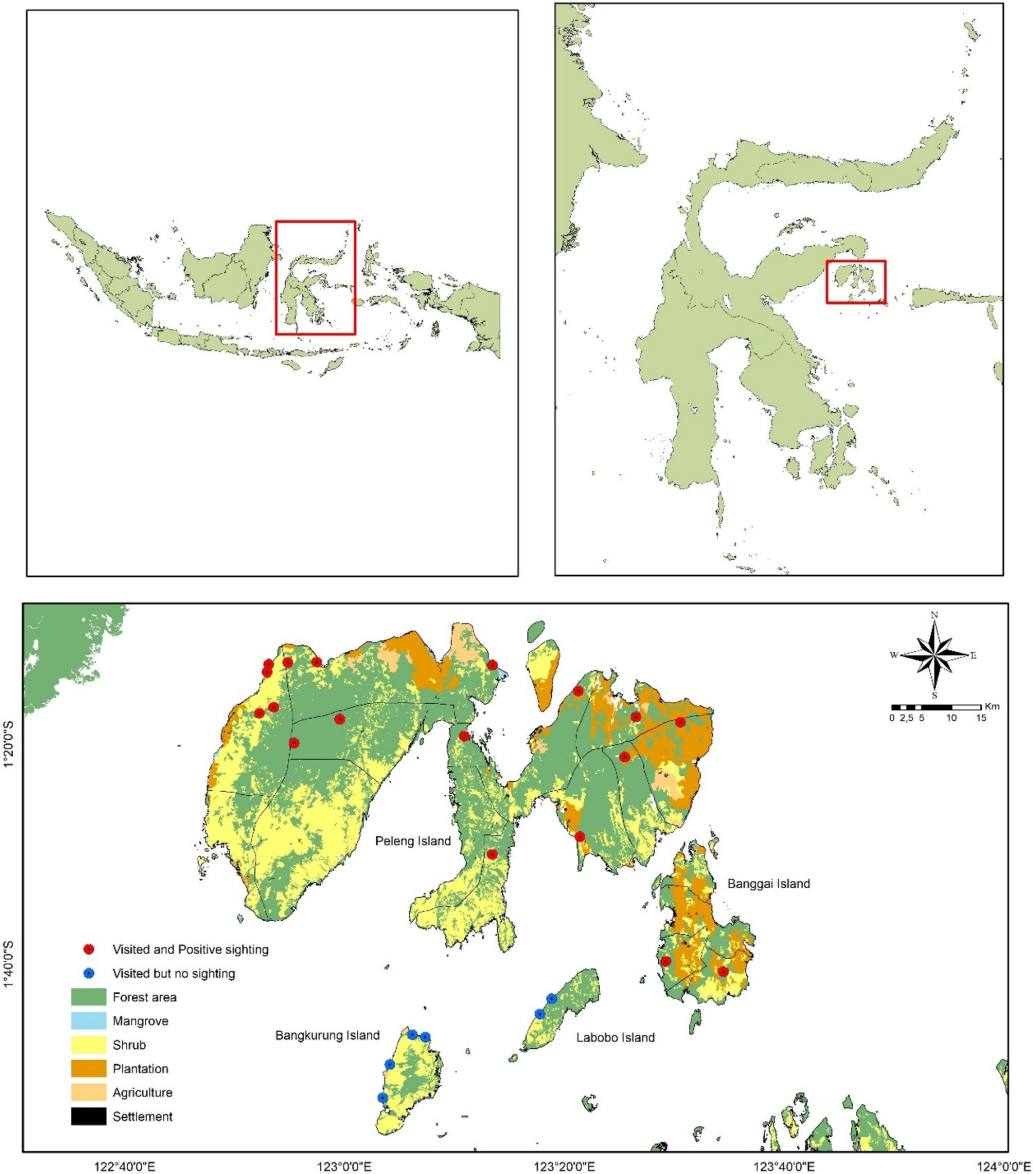
Map of the study area, depicting main land use. Source: Qgis 3.22.14. https://www.qgis.org/en/site/.

This field study was conducted on the four main islands in the Banggai archipelago (Peleng, Banggai, Labobo, and Bangkurung) (Fig. 1).

**Figure 2:**
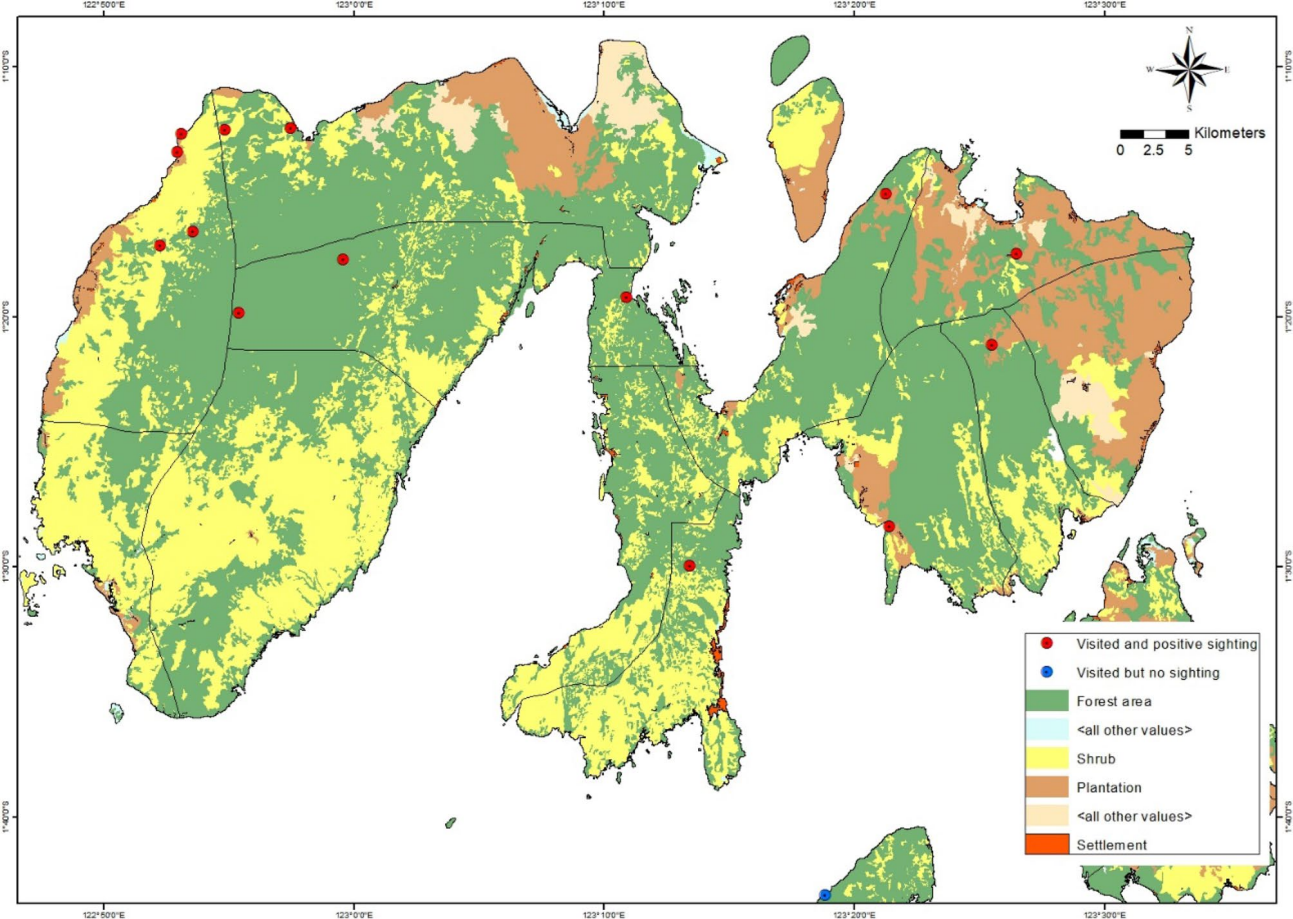
Primary land uses on Peleng island. Source: Qgis 3.22.14. https://www.qgis.org.

**Figure 3:**
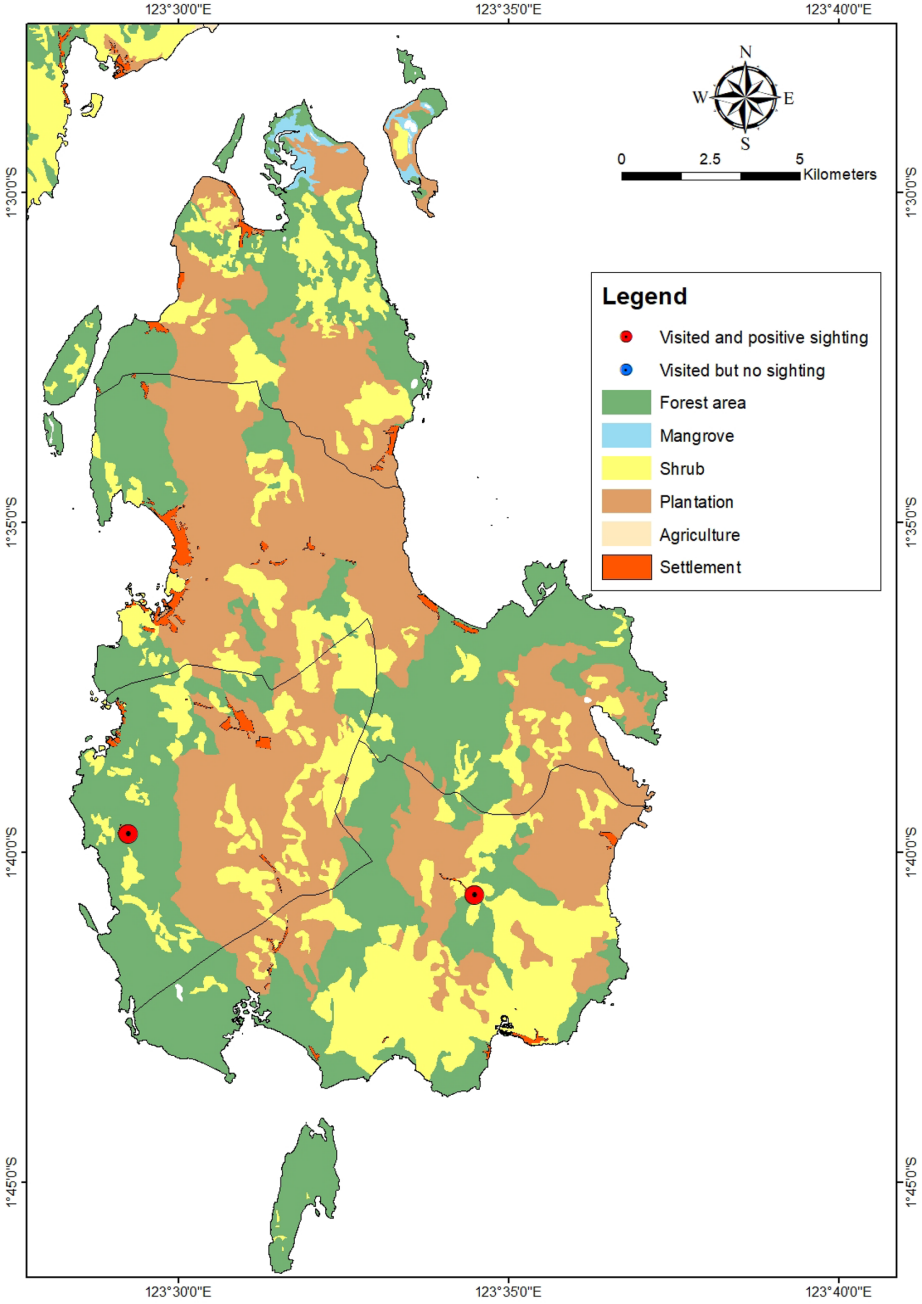
Primary land uses on Banggai Island. Source: Qgis 3.22.14. https://www.qgis.org.

The islands of Peleng, Banggai, Labobo, and Bangkurung, are populated by respectively 121,684, 43,712, 6070, and 8940 humans. There is more natural forest in Peleng I than Banggai I (Fig. 2 and Fig. 3). The detail about location and the coordinate of the study area (Fig. 1) can be found as Supplementary Table S1 online.

### Study methods

A 3-month (Dec. 2016–March 2017) preliminary study focusing on tarsier distribution was conducted on Peleng Island. Habituation was done by conducting daily visitations and staying near the vicinity of certain tarsier localities over the 3 month period. Tarsiers varied in their level of habituation. The tarsiers in Banggai responded to playback calls of the tarsiers from Peleng, and for this reason we are treating them as the same taxonomic unit. Nevertheless, confirmation by morphological and genetic studies is still needed.

#### Tarsier demographic surveys

Primary data were systematically collected from April 7–23, 2017, July 18–September 18, 2017 and March 19–April 19, 2018 in Peleng Island and Banggai, Labobo, and Bangkurung from April 20 until May 19, 2018 for a total of 225 h of contact. The population was sampled using traditional transect methods and analyzed using distance sampling^9^. Transects were walked from 04:00 to 07:00, and 17:00–20:00 h.

For the data collection in Peleng I., transect lines were established in both undisturbed and disturbed forest, and then divided into 5 sections based on altitude:

1. 0–200 m.a.s.l
2. 200–400 m.a.s.l
3. 400–600 m.a.s.l
4. 600–800 m.a.s.l
5. 800–1000 m.a.s.l

However, in Banggai all the transects are lumped together as we did not conduct enough transects there to enable habitat/altitudinal categorization.

Data collected while walking transects included: the number of animals/groups, perpendicular distance, and tarsier activity. To get accurate data, it is necessary to fulfill the assumptions of the Distance method^9^.

1. Animals are distributed independently of the transect.
2. Objects on the line are detected with certainty.
3. Precise distance measurement.
4. Object is detected at its initial location.

If these assumptions are not fulfilled, the results obtained tend to be overestimated. In observing tarsiers, we assume that all animals that are close to the transect are detected, while animals further from the transect will be more difficult to detect. This is represented by the detection function g(x)^9^.

The transects were established using stratified random sampling. Twenty-five transects were walked in Peleng, Banggai, Labobo, and Bangkurung Islands, transects were each walked 3–6 times. The length of transect used was 800 m to 3500 m, and the total transect length was 74.1 km. The total area observed was about 10.01 km^2^. About 20 villages locations across the islands (Peleng, Banggai, Labobo, and Bangkurung) were covered (Table 2).

**Table 2:** Transects used in tarsier surveys.

Island	Total transect	Total length (km)	Total coverage (km^2^)	Person hours effort*
Peleng island	19	62.6	9.16	171 h
Banggai island	2	3.4	0.85	18 h
Labobo	2	3.9	0.97	18 h
Bangkurung	2	4.2	1.05	18 h
Total effort				225 h

^*^Person hours refer to amount of time taken to walk transects by author. At the same time, co author contributed the same effort during the transect walks, thus doubling the table.

#### Distance based detection probability

Each group and individual encountered during the transects were tallied which, yielded encounter rates, densities, and detection probability using software Distance 7.3^9,10^, using CDS (*Conventional Distance Sampling*). The model’s choice for the detection function was based on the smallest AIC (*Akaike’s Information Criterion*) value and the value of ΔAIC = 0^10^. Hazard rate with simple polynomial was selected as the primary function. Minimum AIC value was 344.13.

Distance software show that minimum AIC and Delta AIC equal to zero is the best model for the detection probability. From Table 3, hazard rate with simple polynomial adjustment is the best model.

**Table 3:** Comparison analysis model for detection probability.

Name	Delta AIC	AIC	ESW	D	D LCL	D UCL	D CV
Half normal (Cosine adjustment)	0.29	344.41	4.46	2.488	1.595	3.880	2.218
Half normal (Simple polynomial adjustment)	0.29	344.41	4.46	2.807	0.864	9.123	0.301
Half normal (Hermite polynomial adjustment)	0.29	344.41	4.46	2.488	1.595	3.880	0.218
Hazard rate (Cosine adjustment)	0.29	344.41	4.46	2.807	0.864	9.123	0.301
Hazard rate (Simple polynomial adjustment)	0.00	344.13	4.79	2.464	1.557	3.900	0.227
Hazard rate (Hermite polynomial adjustment)	0.29	344.41	4.46	2.471	1.771	3.448	0.159
Uniform (Cosine adjustment)	1.20	345.33	5.02	2.245	1.468	3.432	0.206
Uniform (Simple polynomial adjustment)	1.65	345.78	4.93	2.690	2.054	3.521	0.137
Uniform (Hermite polynomial adjustment)	1.65	345.78	4.93	2.293	1.488	3.534	0.211

*ESW* effective strip width, *D* density of individual, *D LCL* density of individual analytic lower conf limit, *D UCL* density of individual analytic upper conf limit, *D CV* density of individual analytic coefficient of variance.

#### Habitat description work

We also conducted a vegetation survey to understand the characteristic of the tarsier habitat. We used plotless sampling with point centered quarter method^11^ during which only trees with diameters of more than 30 cm within the plot were recorded. Despite targeting the large trees in our vegetation survey, we found that Peleng tarsiers are using smaller girthed trees and therefore our habitat description is coarse.

The observation points were placed on each transect where tarsiers were encountered and observed. A total of 50 points of vegetation observation were sampled. Data collected by this study include: tree species, diameter at breast height (DBH), tree height (m), and distance between nearest neighbor trees (m). Herbarium specimens were prepared in the field, for later identification by Mr. Ismail A Rahman, a botanical expert from Herbarium Bogoriense, Bogor, Indonesia. The plant data collected was analyzed to find the density, relative density, frequency, relative frequency, abundance, and relative abundance. Relative indexes were used to calculate the importance values index of the species.

## Results

This study established that Peleng tarsiers are geographically distributed in Peleng and Banggai. The Peleng tarsier was not found on either Labobo or Bangkarung.

**Figure 4:**
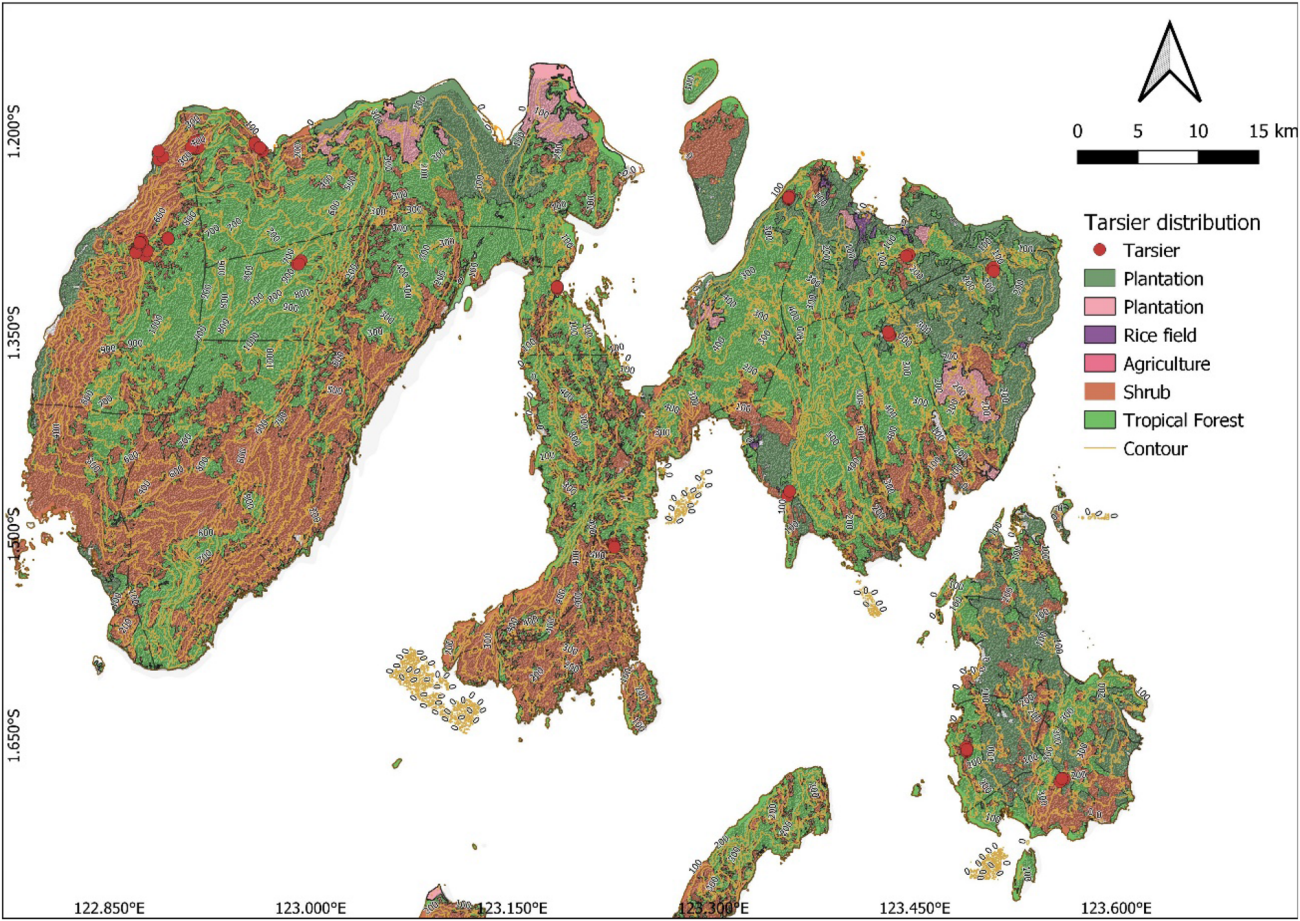
Map of tarsier distribution. Source: Qgis 3.22.14. https://www.qgis.org.

Peleng tarsiers were encountered in nearly every habitat including secondary forests and the home gardens in villages including: Tatendeng, Leme Leme Darat, Okulo Potil, Kokolomboi, and Komba komba (Fig. 4).

The transects yielded direct sightings of 172 individuals and 13 individuals, respectively, in the islands of Peleng and Banggai. The estimated densities of Peleng tarsier in Peleng island was 234 individuals/km^2^ ranging from 148 to 371 individual/km^2^. Coefficients of variation of the density was 22.92% and the effective width of the transect was 4.79 m. A comparison of the density estimate for each island can be found in Table 4.

**Table 4:** Density estimate comparison.

		Estimate	%CV	df	95% Confident interval	
Banggai	DS	2.7822	83.95	1.03	0.4790E − 03	16,158
	D	4.9591	84.19	1.04	0.10430E − 02	23,579
Peleng	DS	1.3156	22.04	28.57	0.84253	2.0543
	D	2.3450	22.92	22.28	1.480	3.7155

*%CV* coefficient of variance, *df* degree of freedom, *DS* density of cluster, *D* density of animals.

**Figure 5:**
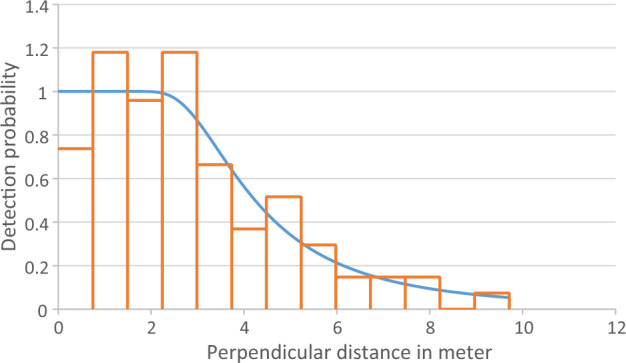
Tarsier survey detection probability.

Based on the software Distance, detection probability g(x) of this study was 28.1%. However, if we truncated the data from 5% (to remove outliers), the detection probability rises to 49.3% (Fig. 5).

The results of the Distance software analysis indicated that the number of group members ranged from 1.7 to 2.01 individuals. Coefficients of variation of cluster size was 6.22%. However, that result was below the direct observations (2–7 individuals).

Our habitat study indicated the occurrence of distinct plants communities corresponding to specific altitude. The less disturbed forest tends to occur above 600 m and as high as 800 m.a.s.l. Vegetation analysis of the primary and secondary forest habitats yielded 69 species of plants from 36 families. Table 5 shows the three highest importance value index (IVI) based on altitudinal elevation.

**Table 5:** Important plants of tarsier habitat, across the altitudinal gradient in Peleng Island.

Local name	Family	Scientific name	RD (%)	RF (%)	RA (%)	IVI (%)
Altitudinal range 0–200 m asl (predominantly secondary forest)						
Kayu tombos	Myrtaceae	*Syzygium glaucum* (King) Chantaran. & J. Prain	15	11.1	48.8	74.9
Bitaul	Clusiaceae	*Calophyllum soulattri* Burm.f	5	5.56	11.1	21.7
Tambade	Sapindaceae	*Mischocarpus sundaicus* Blume	7.5	8.33	2.76	18.6
Altitudinal 200–400 m asl (predominantly secondary forest)						
Sosom	Burseraceae	*Canarium asperum* Benth	15	9.68	12.87	38
Pilapuk	Elaeocarpaceae	*Elaeocarpus sp*	7.5	9.68	18.87	36
Tambade	Sapindaceae	*Mischocarpus sundaicus* Blume	10	9.68	8.35	28
Altitudinal range 400–600 mdpl (predominantly secondary forest)						
Osa	Fagaceae	*Castanopsis acuminatissima* (Blume) A. DC	17.5	11.76	23.2	52.4
Onik	Dipterocarpaceae	*Shorea selanica* (Lam.) Blume	10	8.82	17.1	36
Suloi	Fagaceae	*Lithocarpus celebicus* (Miq.) Rehder	10	8.82	10.8	29.6
Altitudinal range 600–800 m asl (predominantly primary forest)						
Osa	Fagaceae	*Castanopsis acuminatissima* (Blume) A. DC	10	6.06	25.37	41.4
Kanar	Myrtaceae	*Syzygium fastigiatum* (Blume) Merr. & L.M. Perry	20	12.1	4.22	36.3
Takios	Lauraceae	*Cryptocarya glauca* Merr	2.5	3.1	27.06	33.1
Altitudinal range 800–1000 m asl (predominantly primary forest)						
Onik	Dipterocarpaceae	*Shorea selanica* (Lam.) Blume	5	6.06	23.76	34.8
Suloi	Fagaceae	*Lithocarpus celebicus* (Miq.) Rehder	10	9.1	12.1	31.2
Kanar	Myrtaceae	*Syzygium fastigiatum* (Blume) Merr. & L.M. Perry	7.5	9.09	12.42	29

*RD* relative density, *RF* relative frequency, *RA* relative abundance, *IVI* importance value index.

## Discussion

Encounter rates for Peleng tarsiers on Peleng island and Banggai islands was 1.34 and 2.66 individuals/km^2^, respectively. The latter estimate however may be confounded by smaller sample size. For instance, the Distance generated Coefficient of Variation from the encounter rate in Banggai island was 85.3%. Nevertheless, the rate of encounters in Banggai island was 3–9 individuals per km^2^. Since Banggai Islands has a modest forest coverage (Table 2), the figure seems to confirm that Peleng tarsiers can and do colonize degraded habitats, suggesting it is less endangered than previously believed. The calculated Peleng tarsier density in Peleng Island of 234 individuals/km^2^ was relatively high (Table 6). Our tarsier detection rate in the field was 1–5 groups/km^2^, or 2- 15 individuals/km^2^. This relative abundance range appeared to be consistent with the Density-generated result.

**Table 6:** Estimated known densities of different tarsier species and Peleng tarsier.

No	Species	Density
1	*Tarsius dentatus*	45–400 individuals/km^2^^12,13^
2	*T pumilus*	92 individuals/ 100 ha^14^
3	*T pelengensis*	234 individuals/km^2^ (this study)
4	*T lariang*	80.21 ± 30.1 individuals/Km^2^ in primary forests; 218.29 ± 134.59 individuals/Km^2^ in secondary forests^15^
5	*T spectrumgurskyae*	156 individuals/km^2^^16^

The detection curve showed that approximately 28.1% of the whole population was detected by the transect walks. This might happen because the tarsier moves before being recorded and detected. For now, we cannot confirm that we had overestimated Peleng tarsier density. While the detection probability during this study was 28.1%, when we truncated the data from 5% (outliers), we increase the detection probability to 49.3%.

We are fairly confident that we minimized missing detecting tarsiers, for three reasons. Firstly, the maximum group sizes (7 individuals in a travelling cluster) is on par with other tarsier species mentioned. Second, the population sampling is guided by the second author (Un Maddus, a former hunter) who has monitored tarsier distribution before and after the six months’ study period. Third, we found on 10 occasions that tarsier is attracted to low light flash lights, and this compensated or decreased our chances of missing detection.

### Peleng tarsier known distribution

Our field survey failed to discover Peleng tarsier in the neighboring island of Labobo and Bangkurung. However, more studies are needed to establish the Peleng tarsier distribution beyond Peleng and Banggai islands.

### Tarsier demographic surveys

Our method differed with Gursky^16^ and Merker & Mühlenberg^17^, whom used, respectively, fixed point count to estimate spectral tarsier densities and quadrat census distance to density conversion to estimate Dian’s tarsier densities. Gursky’s^16^ modified form of the fixed-point count and quadrat census method computed total number of groups of *Tarsius spectrum* present in each hectare and this allows an estimate of the density for the sampled area. The plots were chosen randomly within the 1 km^2^ using random block design^16^.

In Merker and Mühlenberg’s^18^ use of distance to density conversion, the number and spacing of tarsier family sleeping trees were considered to be a measure of habitat quality. Once all the sleeping trees in each habitat were known, the distance to their three nearest neighbor group were measured. The subsequent mapping of all sleeping sites resulted in estimates of population densities^12,17^. Among these studies none of the methodology has been standardized including this study. However, this study is quite detailed.

There are assumptions made when using distance sampling. Objects are detected with certainty, object do not move, and measurements are exact^10^. Distance sampling requires large number of transect and randomized locations of transects. If the assumptions are not fulfilled biases will happen. In our case, the most likely source of bias is caused by failure in detection before moving. There is also the likelihood that the transects on the neighboring islands of Banggai, Labobo, and Bangkurung were too few to provide concrete results.

### Tarsier behavior ecology

In our field observation, tarsiers traveled either alone or in groups of two to seven, whereas as many as nine individuals were observed at one sleeping site. The group size of 2–7 individuals, was similar to Dian’s Tarsier group size^12,18,19^. Groups were observed to use one—three sleeping trees on alternate days, whereas use of as many as five alternate sleeping trees have also been observed, possibly due to human disturbance. A similar behavior was noted in mainland Sulawesi, the tarsier frequently retreat to an alternate shelter when disturbed^13^. Based on our preliminary study they foraged from 3 to 10 m above. Cursory observations of the tarsier in Peleng suggested that male and female ranged over areas of 10 ha and 8 ha, respectively. In mainland Sulawesi, the male tarsier moves further than the females^20^. With the western tarsier *Tarsius bancanus*^21^, the male range was 11.25 ha while the female home range about 4.5 ha. Peleng tarsiers perform vocal duets at dawn before going to their sleeping site and infrequently at dusk. Tarsier species are known to regularly vocalize, and emit duets. In comparison, *Carlito* call less and *Cepalopachus* even less, and none of them are known to duet^1^.

### Group size

The group sizes of 2–7 individuals noted in the field (Peleng) were higher than the result of the Distance analysis (1.7–2 individuals in a group). During the transect not all the individuals were detected and this might be a reason why Distance-generated estimates had smaller group sizes compared to the direct encounters.

In other tarsier species, the groups are showing various sizes, but generally in the range of Peleng tarsier’s, i.e. the larger travelling groups ranged from five to seven. The group size of pygmy tarsier, *Tarsius pumilus*, was from two to five individuals^14^ whereas Dian’s tarsier group live in small family of up to seven individuals^19^ and the western tarsier group size was reported two to eight individuals^22^.

### Conservation

Naturally, tarsier density is affected by various factors such as vegetation, predators, availability of food, and disturbance^12,23^. In Peleng, tarsiers were more common in secondary forests than in primary forests. With the western tarsier, *T. bancanus*, it was also noted that sapling and poles were more often utilized compared to large trees, (Ref.^21^. This is unsurprising in that previous studies (e.g.^18^.) have observed that tarsiers are more abundant in secondary forests, in recently logged forests, shrubs, and bamboo or secondary forests with small-scaled disturbances^24–26^.

Forest fires and land clearing by burning occurred regularly in Peleng, and caused deforestation and degradation of habitat. Besides fisheries, subsistence agriculture is one of the primary occupations of the local community. The main agricultural commodities were tubers, and some agroforestry produces such as peanut (*Arachis hypogea*), cashew (*Anacardium occidentale*)*,* fragrant nutmeg (*Myristica fragrans*)*,* clove (*Syzygium aromaticum*). Intensive agriculture as a matter of course can reduce the species richness in the mosaic of the forested landscape^27^. Further, intensive land use may decrease mammalian density especially on larger sized animals^28^. In Peleng, wildlife-threatening forest fires occurred in the dry season in 2015, within the boundaries of Okulo Potil village (Yoris Yosukule pers. comm, 31 Oct 2017). For the local community, use of fire in agriculture is the easiest, quickest, and cheapest way to clear land (see also Ref.^29^). While forest fires are known to occur regularly and at least cause temporary local extinction of the tarsier population, they have not resulted in the extinction of the population throughout the island.

We currently do not have information concerning whether food is a limited factor for the Peleng tarsier population. Peleng tarsiers prey on insects such as grasshopper (Orthoptera), fireflies (Coleoptera), and spiders (Aranae) (Alpian Maleso, pers comm, 24 Aug 2017). In mainland Sulawesi, tarsiers were known to eat insects from the orders of Aranae, Coleoptera, Isoptera, Homoptera, Hymenoptera, Lepidoptera, and Orthoptera^20^. The western tarsier *T. bancanus*, was even known to feed on small snakes and lizards^21,24,30^. Foraging behavior and diet preferences of some tarsier species are known to be affected by seasonality. In north Sulawesi, during the rainy season, the tarsier’s dietary preferences differ relative to the dry season. In the rainy season, Gursky’s spectral tarsiers tend to hunt Orthoptera and Lepidoptera^20^. *Tarsius pumilus*, the high altitude tarsier, primarily consumes insects found in secondary vegetation along the forest edges^14^.

Some tarsier species in Sulawesi and in the Philippines tarsiers were predated upon by snakes, lizards, owls, eagles, cats, civets^31^, with *Python reticulatus* as a potentially important predator^32^. Python, lizards, eagle, owl, civet also occur on Peleng and thus are also potential tarsier predators. During our observations, the common *Spilornis rufipectus,* Sulawesi Serpent-eagle, was stealthily watching a lone tarsier (Un Maddus personal observation). However, no observations of a tarsier being preyed upon were made during our study.

The current conservation status of the Peleng tarsier, based on IUCN criteria^33^ is Endangered (EN) B1ab(iii). This means that the geographic distribution is smaller than 5000 km^2^, the density estimated show serious fragmented population or could be found only at one location and decreasing area or habitat quality^34^. Gursky and colleagues^3^ suggested that suitable habitat for tarsiers on Peleng was only 10% of the island based on assumption of habitat extent, but not based on any actual field survey.

However, based on our recent field study, the total population of the Peleng tarsier in our study area is estimated to be 2270 individuals from 1001 Ha. Given the relatively high density of tarsiers in forested habitat on Peleng, and their ability to inhabit human modified areas, they do not meet the Endangered criteria B1ab (iii). Hence, we propose that the conservation status of the Peleng tarsier should be changed to “Vulnerable”.

The Peleng tarsiers are not hunted and are found on both Peleng and Banggai islands. Increased habitat protection mainly by communities as part of longer-term local people-centered capacity building^35^ has been an effective strategy. In fact, the local community has independently conducted their own wildlife conservation outreach, and a growing number of community-conserved areas have been established in the villages of Kokolomboi/Lemeleme Darat, Komba-komba, Kawalu/Kautu, and Batong. Given the investment of the local community into the conservation of the Peleng tarsier, we are very hopeful that this species will continue to thrive and its conservation status continually improved.

## Conclusions

Peleng tarsiers were encouragingly found across highly varied habitat types, and were estimated to have a total density of 234 individuals/km^2^ in our study area. Our field data demonstrated clear abundance and adaptation to high levels of human disturbances throughout the two islands. Consequently, we would like to suggest the Peleng tarsier to be declassified from Endangered status category (Supplementary Table).

## Supplementary Information

Supplementary Information.

## Data Availability

The datasets generated and/or analysed during the current study are available from the First author (FNS) on reasonable request.
