# Supplementary material for: Distribution and abundance of Peleng Tarsier (Tarsius pelengensis) in Banggai Island group, Indonesia
Source: Sci Rep. 2023 Jul 15;13:11445. doi: 10.1038/s41598-023-30049-5 (PMC10349819; doi:10.1038/s41598-023-30049-5)
Supplement: Supplementary file 1 — Supplementary Information. [file 41598_2023_30049_MOESM1_ESM.docx]

**Geographic coordinate of study site**

| **Positive sighting** | | |
| --- | --- | --- |
| **Peleng Island** | | |
| Bolubung | 123° 13' 29,462" E | 1° 12' 45,944" S |
| Lukpanenteng | 122° 57' 30,481" E | 1° 12' 28,584" S |
| Lipubasal | 122° 59' 35,599" E | 1° 17' 41,978" S |
| Tatendeng | 122° 53' 35,110" E | 1° 16' 35,184" S |
| kokolomboy | 122° 52' 16,882" E | 1° 17' 8,318" S |
| Okulo potil | 122° 52' 58,930" E | 1° 13' 23,920" S |
| Olusi | 122° 53' 8,340" E | 1° 12' 40,763" S |
| Pondok yoris | 122° 54' 51,898" E | 1° 12' 31,324" S |
| Mbumbu | 122° 55' 25,766" E | 1° 19' 51,398" S |
| Komba Komba | 123° 10' 54,826" E | 1° 19' 13,062" S |
| Nosuon | 123° 30' 34,222" E | 1° 17' 57,192" S |
| Basosol | 123° 13' 27,761" E | 1° 29' 58,175" S |
| Polintang | 123° 21' 25,294" E | 1° 28' 22,804" S |
| Mata | 123° 25' 31,217" E | 1° 21' 7,196" S |
| Bangpanga | 123° 26' 30,804" E | 1° 17' 29,346" S |
| Kawalu | 123° 21' 17,334" E | 1° 15' 5,728" S |
| **Banggai Island** | | |
| Posolalongo | 123° 29' 15,284" E | 1° 39' 42,617" S |
| Malino | 123° 34' 29,471" E | 1° 40' 38,554" S |
| **Negative sighting** | | |
| **Labobo Island** | | |
| Lintolu | 123° 18' 51,872" E | 1° 43' 6,337" S |
| Padingkian | 123° 17' 46,716" E | 1° 44' 30,394" S |
| **Bangkurung Island** | | |
| Dungkean | 123° 6' 11,524" E | 1° 46' 23,394" S |
| Beleang | 123° 3' 29,761" E | 1° 52' 7,111" S |
| Lenggetan | 123° 4' 8,580" E | 1° 49' 6,978" S |
| Saboa | 123° 7' 22,602" E | 1° 46' 35,339" S |
